# Paclitaxel and curcumin coadministration in novel cationic PEGylated niosomal formulations exhibit enhanced synergistic antitumor efficacy

**DOI:** 10.1186/s12951-018-0351-4

**Published:** 2018-03-23

**Authors:** Ashraf Alemi, Javad Zavar Reza, Fateme Haghiralsadat, Hossein Zarei Jaliani, Mojtaba Haghi Karamallah, Seyed Ahmad Hosseini, Somayeh Haghi Karamallah

**Affiliations:** 1 Department of Clinical Biochemistry, Faculty of Medicine, Shahid Sadoughi University of Medical Sciences, Yazd, Iran; 2 Biotechnology Research Center, International Campus, Shahid Sadoughi University of Medical Science, Yazd, Iran; 3 Department of Life Science Engineering, Faculty of New Sciences & Technologies, University of Tehran, Tehran, Iran; 4 Protein Engineering Laboratory, Department of Medical Genetics, School of Medicine, Shahid Sadoughi University of Medical Sciences, Yazd, Iran; 5 Nutrition and Metabolic Diseases Research Center, Ahvaz Jundishapur University of Medical Sciences, Ahvaz, Iran; 6 Student Research Committee, Shiraz University of Medical Sciences, Shiraz, Iran

**Keywords:** Niosome, Paclitaxel, Curcumin, Combination therapy, Chemotherapy

## Abstract

**Background:**

The systemic administration of cytotoxic chemotherapeutic agents for cancer treatment often has toxic side effects, limiting the usage dose. To increase chemotherapeutic efficacy while reducing toxic effects, a rational design for synergy-based drug regimens is essential. This study investigated the augmentation of therapeutic effectiveness with the co-administration of paclitaxel (PTX; an effective chemotherapeutic drug for breast cancer) and curcumin (CUR; a chemosensitizer) in an MCF-7 cell line.

**Results:**

We optimized niosome formulations in terms of surfactant and cholesterol content. Afterward, the novel cationic PEGylated niosomal formulations containing Tween-60: cholesterol:DOTAP:DSPE-mPEG (at 59.5:25.5:10:5) were designed and developed to serve as a model for better transfection efficiency and improved stability. The optimum formulations represented potential advantages, including extremely high entrapment efficiency (~ 100% for both therapeutic drug), spherical shape, smooth-surface morphology, suitable positive charge (zeta potential ~ + 15 mV for both CUR and PTX), sustained release, small diameter (~ 90 nm for both agents), desired stability, and augmented cellular uptake. Furthermore, the CUR and PTX kinetic release could be adequately fitted to the Higuchi model. A threefold and 3.6-fold reduction in CUR and PTX concentration was measured, respectively, when the CUR and PTX was administered in nano-niosome compared to free CUR and free PTX solutions in MCF-7 cells. When administered in nano-niosome formulations, the combination treatment of CUR and PTX was particularly effective in enhancing the cytotoxicity activity against MCF-7 cells.

**Conclusions:**

Most importantly, CUR and PTX, in both free form and niosomal forms, were determined to be less toxic on MCF-10A human normal cells in comparison to MCF-7 cells. The findings indicate that the combination therapy of PTX with CUR using the novel cationic PEGylated niosome delivery is a promising strategy for more effective breast cancer treatment.

## Background

Chemotherapy is the standard treatment for various types of cancers. However, chemotherapy is associated with high systemic toxicity and low therapeutic effectiveness [1]. Nanotechnology has revolutionized the diagnosis and treatment of cancer [2]. A nano-sized drug delivery system (DDS), or nanocarrier, is designed to deliver therapeutic and/or diagnostic agents to their target sites [3]. Over recent decades, drug delivery systems using vesicular carriers have attracted great interest because these carriers provide high encapsulation efficiency, control drug release, enhance drug solubility, carry both hydrophilic and hydrophobic drugs, reduce side effects, prolong circulation in blood, and possess the ability to target a specific area [4, 5]. Vesicles made of natural or synthetic phospholipids are called liposomes, while transferosomes are modified liposomal systems that, in addition to phospholipids, contain a single chain surfactant as an edge activator; ethosomes contain ethanol as an edge activator instead of a single chain surfactant. Despite having some advantages over conventional dosage forms, vesicular carriers present many problems in practical applications, such as high cost, the use of organic solvents for preparation, and a limited shelf life due to lipid rancidification [6]. Therefore, a continuous endeavor has been made to find an alternative vesicular carrier. Niosomes meet this requirement [7]. Niosomes, or non-ionic surfactant vesicles, are unilateral or multilamellar spheroidal structures. Niosomes are preferred as an effective alternative to conventional liposomes, as they offer several advantages, including greater stability, lower cost, biodegradability, biocompatibility, non-immunogenic, and low toxicity, and they can be stored more easily for industrial production in pharmaceutical applications [5, 8–12]. To improve stability and circulation half-life, niosomes may be coated with appropriate polymer coatings, such as polyethylene glycol (PEG), creating PEGylated niosomes. PEG coating also helps reduce systemic phagocytosis, which results in prolonged systemic circulation, as well as reduced toxicity profiles [13, 14]. Paclitaxel (PTX) is an important antineoplastic drug, and it is isolated from the bark of *Taxus brevifolia*. PTX demonstrates an effective chemotherapeutic and cytotoxic activity against breast, ovarian, colon, lung, prostate, and brain cancers. However, the wide therapeutic effects of PTX are limited due to the low therapeutic index and poor water-solubility [15, 16]. Curcumin (CUR) is a hydrophobic polyphenol compound obtained from the rhizome of the plant *Curcuma longa*. CUR exhibits various pharmacological activities, such as anti-inflammatory, anti-oxidant, and anti-tumor effects. Particularly, CUR has been demonstrated to be highly effective against a variety of different malignancies, including leukemia and lymphoma, as well as colorectal, breast, lung, prostate, and pancreatic carcinoma. However, the pharmacological application of CUR has been impeded due to its extremely low aqueous solubility, instability, extremely poor bioavailability, and high metabolic rate [17–19]. As a result, nanotechnology is considered one of the most significant methods to design and develop various nano-carrier formulations for curcumin and paclitaxel, such as polymeric micelles, liposomes, self-assemblies, nanogels, niosome biodegradable microspheres, and cyclodextrin [18, 20, 21]. In this study, we loaded both curcumin and paclitaxel into cationic PEGylated niosomal formulations for enhanced efficacy in MCF-7 human breast adenocarcinoma cells. In addition to formulation design and optimization, we have examined release profile, intracellular delivery, and enhancement of cytotoxicity appears.

## Results

### The effect of surfactant:cholesterol ratio on CUR/PTX niosome formulations

To specify the optimal formulation for attaining high entrapment efficiency, controlled release (at 37 °C and pH 7.4), and small vesicle size, various niosomal CUR/PTX formulations were evaluated (Table 1). As shown in Table 1, cholesterol had a profound effect on CUR/PTX entrapment efficiency in niosomes: by increasing the amount of cholesterol content from 10% in formulation 1 (F1) to 30% in formulation 4 (F4), PTX/CUR entrapments into nano-niosomes were constantly increased. However, adding cholesterol from F1 to F4 decreased the percentage of CUR/PTX released over 12 h. Furthermore, as can be seen from the presented results, the mean diameter of the niosomes increased with increasing the cholesterol content (F1 → F5, Table 1). However, the addition of up to 50% cholesterol to niosomes in F5 decreased niosomal efficiency in trapping curcumin/paclitaxel compared to the 30% cholesterol content in F4. Based on high entrapment efficiency and sustained drug release, the F4 formula has chosen as the formulation for further studies.

**Table 1:** Effect of the non-ionic surfactant Tween 60: cholesterol with various molar ratios on entrapment efficiency (EE %), size and % release (R) in CUR/PTX loaded Niosomes

Code	Mole Tween 60 (%)	Mole cholesterol (%)	EE (%)	R (%)	Size (nm)	PDI	Zeta potential (mV)
F1	90	10	EE % CUR = 52.24 ± 0.47 EE % PTX = 45.24 ± 0.12	R % CUR = 75.26 ± 0.42 R % PTX = 65.14 ± 0.32	Size CUR = 101.5 ± 0.12 Size PTX = 122.4 ± 0.46	PDI CUR = 0.281 ± 0.056 PDI PTX = 0.261 ± 0.056	Zeta CUR = − 20.34 ± 0.68 Zeta PTX = − 16.62 ± 0.47
F2	80	20	EE % CUR = 66.12 ± 0.86 EE % PTX = 61.74 ± 0.36	R % CUR = 69.11 ± 0.24 R % PTX = 56.12 ± 0.66	Size CUR = 107.5 ± 0.31 Size PTX = 131.24 ± 0.31	PDI CUR = 0.246 ± 0.12 PDI PTX = 0.236 ± 0.12	Zeta CUR = − 22.41 ± 0.75 Zeta PTX = − 19.08 ± 0.36
F3	75	25	EE % CUR = 81.24 ± 0.47 EE % PTX = 72.44 ± 0.63	R % CUR = 57.26 ± 0.11 R % PTX = 47.24 ± 0.36	Size CUR = 112.7 ± 0.64 Size PTX = 140.66 ± 0.72	PDI CUR = 0.224 ± 0.087 PDI PTX = 0.214 ± 0.087	Zeta CUR = − 21.38 ± 0.86 Zeta PTX = − 21.54 ± 0.44
F4	70	30	EE % CUR = 85.42 ± 0.11 EE % PTX = 81.37 ± 0.21	R % CUR = 46.11 ± 0.34 R % PTX = 39.22 ± 0.41	Size CUR = 118.7 ± 0.56 Size PTX = 149.32 ± 0.65	PDI CUR = 0.204 ± 0.062 PDI PTX = 0.194 ± 0.062	Zeta CUR = − 21.45 ± 0.42 Zeta PTX = − 19.56 ± 0.27
F5	50	50	EE % CUR = 71.24 ± 0.16 EE % PTX = 67.12 ± 0.47	R % CUR = 54.12 ± 0.22 R % PTX = 45.14 ± 0.32	Size CUR = 125.1 ± 0.44 Size PTX = 157.44 ± 0.66	PDI CUR = 0.214 ± 0.013 PDI PTX = 0.208 ± 0.013	Zeta CUR = − 24.16 ± 0.22 Zeta PTX = − 24.56 ± 0.42

### The effect of DSPE-mPEG (2000) and DOTAP in niosomal formulation

For attaining less aggregation, smaller niosomes, higher entrapment efficiency, and improved stability, 5% PEG was added to F4. According to Table 2, the F6 niosomal formula containing 5% PEG showed higher drug entrapment, smaller diameter, smaller Poly-Dispersity Index (PDI), and lower drug release than the F4 formula. Table 2 shows the number of positive charge particles and the entrapment efficiency were increased by adding 10% DOTAP to F6. However, vesicle size and PDI declined with a 10% increase in the molar amount of DOTAP. The obtained results showed the CUR/PTX niosomal formulations containing Tween-60: cholesterol:DOTAP:PEG with a 59.5:25.5:10:5 molar ratio (F7) had the desired feature based on high entrapment efficiency, sustained drug release, small diameter, and improved transfection efficiency (Table 2).

**Table 2:** Effect cationic phospholipid DOTAP and DSPE-mPEG (2000) on entrapment efficiency (EE %), size and % release (R) in CUR/PTX loaded Niosomes

Code	Mole Tween 60 (%)	Mole cholesterol (%)	Mole DOTAP (%)	Mole PEG (%)	EE (%)	R (%)	Size (nm)	PDI	Zeta potential (mV)
F6	64.4	27.6	0	5	EE % CUR = 91.22 ± 0.28 EE % PTX = 86.11 ± 0.66	R % CUR = 38.53 ± 0.18 R % PTX = 31.44 ± 0.16	Size CUR = 91.5 ± 0.25 Size PTX = 118.9 ± 0.31	PDI CUR = 0.179 ± 0.23 PDI PTX = 0.164 ± 0.31	Zeta CUR = − 20.99 ± 0.45 Zeta PTX = − 19.24 ± 0.44
F7	59.5	25.5	10	5	EE % CUR = 98.24 ± 0.11 EE % PTX = 98.79 ± 0.24	R % CUR = 33.11 ± 0.33 R % PTX = 20.08 ± 0.44	Size CUR = 85.4 ± 0.43 Size PTX = 111.6 ± 0.24	PDI CUR = 0.177 ± 0.17 PDI PTX = 0.158 ± 0.24	Zeta CUR = + 14.83 ± 0.21 Zeta PTX = + 16.17 ± 0.31

### Physical characterization of niosomal vesicles

**Fig. 1:**
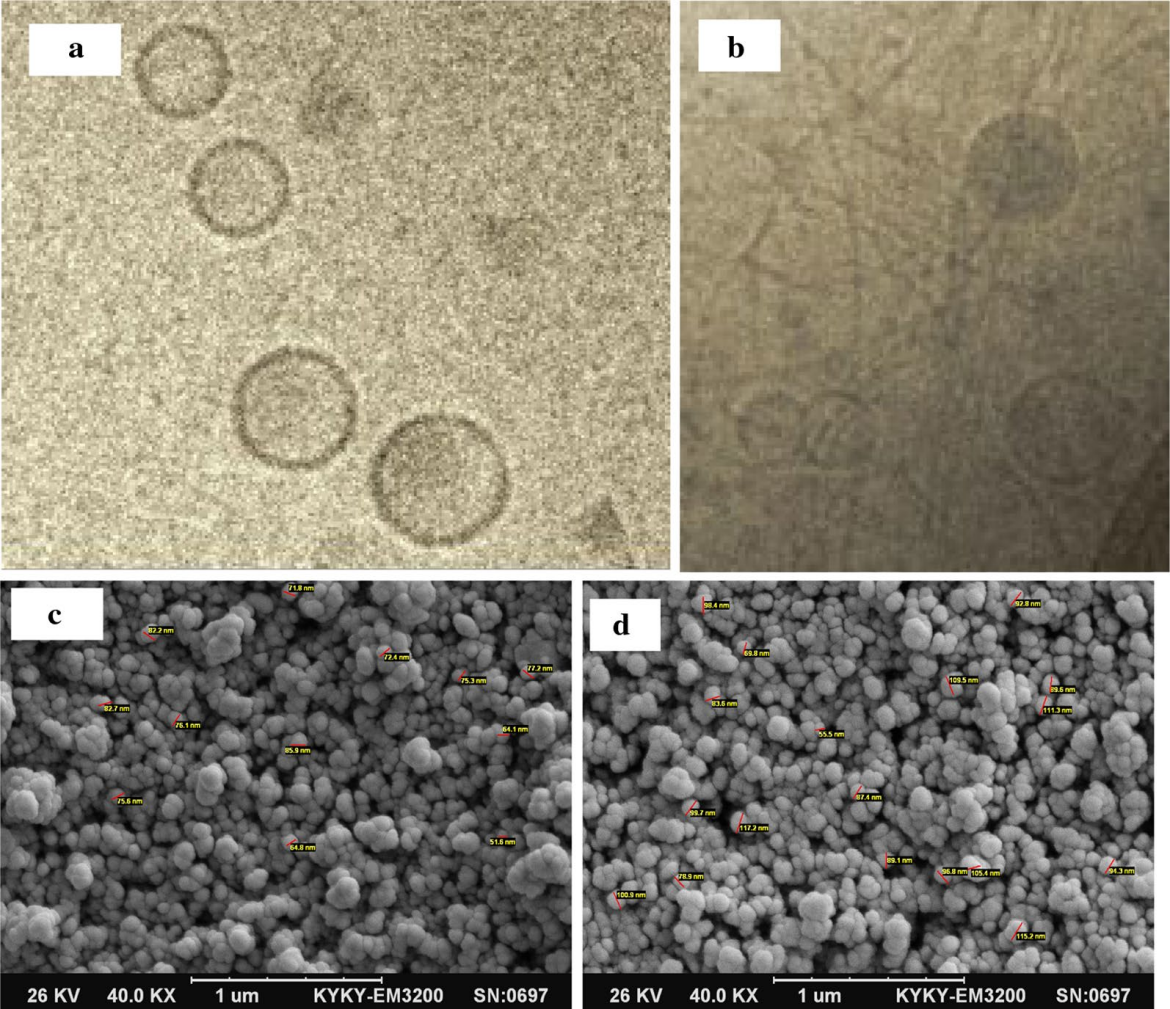
Morphological assessment: **a** niosomal paclitaxel; **b** niosomal curcumin by cryogenic transmission electron microscopy (Cryo-TEM). Scanning electron microscopy (SEM) of **c** curcumin niosome; and **d** paclitaxel niosome

The internal structure of CUR/PTX niosomes was evaluated by cryogenic transmission electron microscopy (Cryo-TEM). As illustrated in Fig. 1a, b, the optimum formula of CUR/PTX niosomes was spherically shaped. Furthermore, the niosomes structures’ rigid boundaries were indicated. According to SEM photographs, the niosomal vesicles were found to be round with smooth surfaces (Fig. 1c, d).

### In-vitro drug release study

**Fig. 2:**
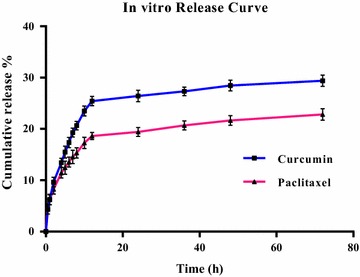
The in vitro release profile of curcumin and paclitaxel from niosomal optimum formula

Evaluation of in vitro drug release was performed using the dialysis method. The results of a 72-h release profile of CUR and PTX from the optimum formulation (F7) in PBS pH 7.4 at 37 °C are displayed in Fig. 2. After 72 h, 29.93 and 28.16% of the loaded drugs were released for CUR and PTX, respectively. The cumulative release profile of CUR and PTX was apparently biphasic, with an initial rapid release period followed by a slower release phase.

### Release kinetics modeling

**Fig. 3:**
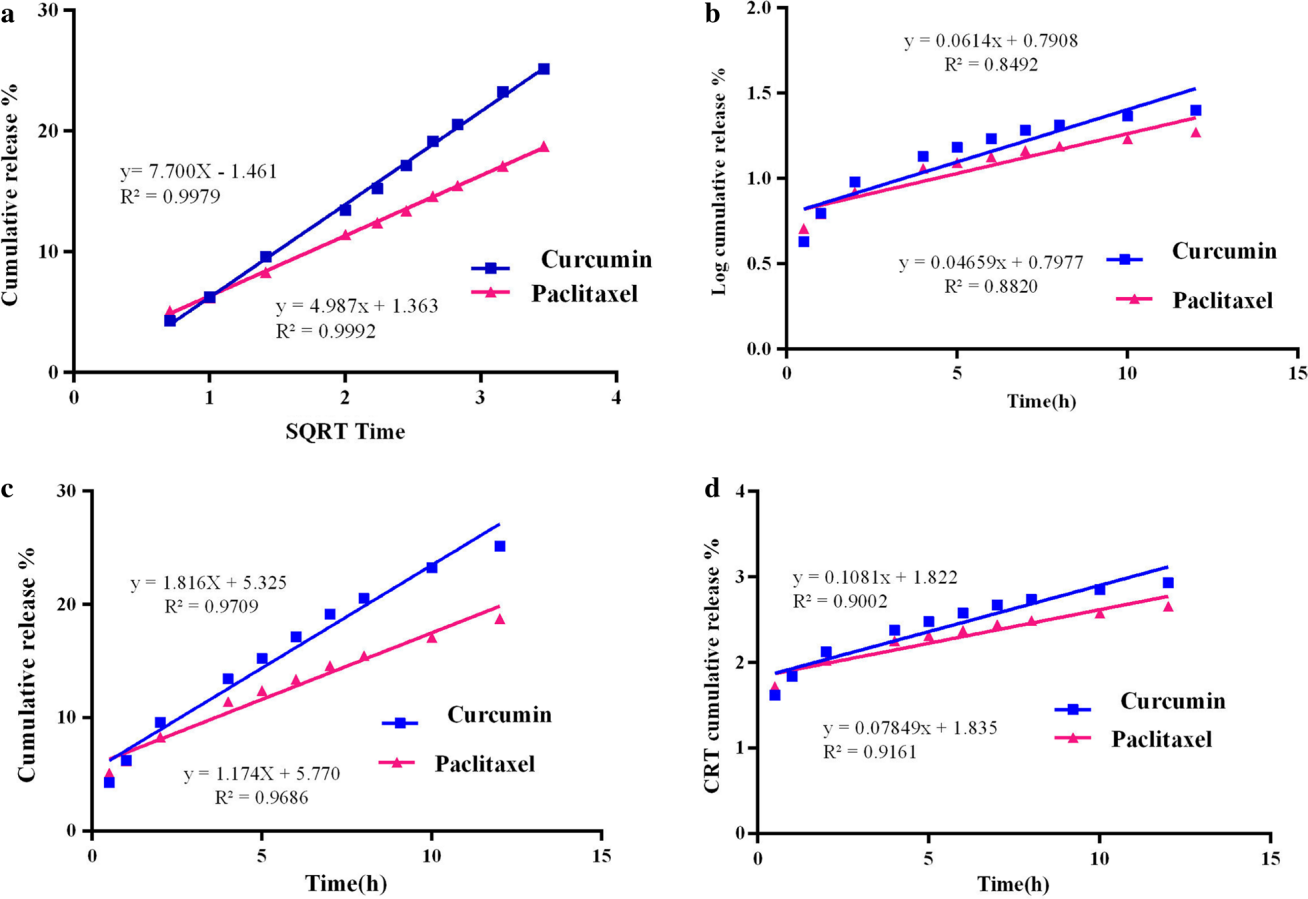
Curcumin and paclitaxel comparative plots. **a** Zero order release kinetics; **b** first order release kinetics; **c** Higuchi (SQRT) release kinetics and **d** Hixson–Crowell model

Figure 3 shows the CUR/PTX release data were analyzed mathematically according to: zero-order, first-order, Hixson–Crowell, and Higuchi’s equations. Table 3 summarizes the correlation coefficients (R^2^) calculated for niosomal formulations. The results revealed that the release of CUR and PTX from niosomal films is most fitted to the Higuchi model, according to the higher correlation coefficient.

**Table 3:** Release kinetics data of CUR and PTX from the niosomal optimum formulae

Regression coefficient (R^2^)				
Formulation code	Zero order	First order	Higuchi model	Hixson–Crowell model
F7 (CUR)	0.9709	0.8492	0.9979	0.9002
F7 (PTX)	0.9686	0.882	0.9992	0.9161

### Fourier transforms infrared (FTIR) spectral evaluation

**Fig. 4:**
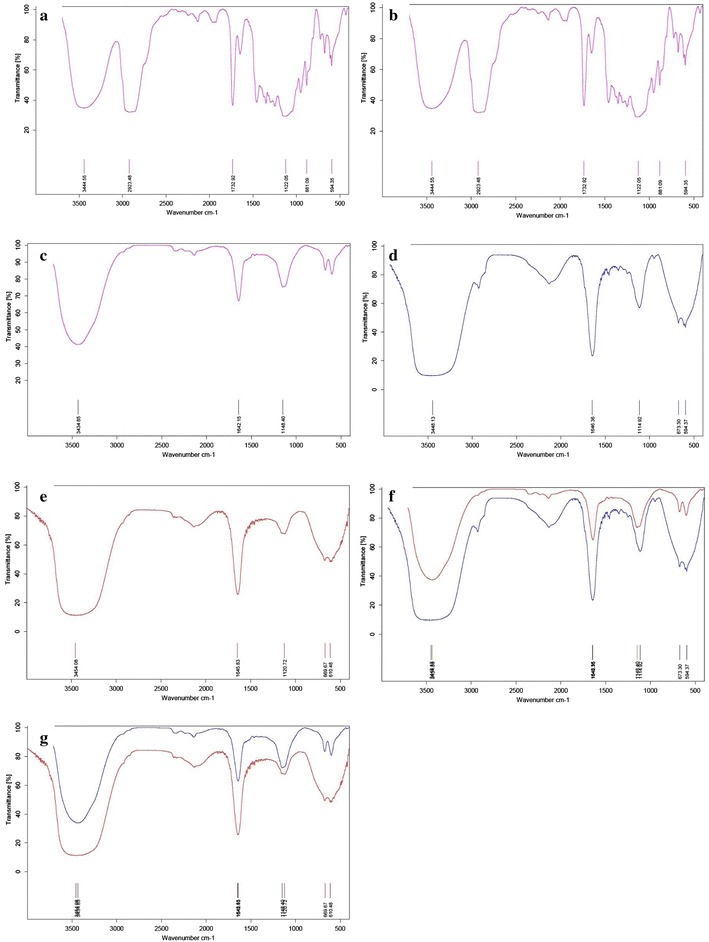
FTIR spectra. **a** Free paclitaxel; **b** free curcumin; **c** blank noisome; **d** niosomal paclitaxel; **e** niosomal; curcumin; **f** comparison blank noisome and niosomal paclitaxel; **g** comparison blank noisome and niosomal curcumin

To confirm the drug presence in CUR/PTX nano-niosome formulations, FTIR analysis was performed. Figure 4a shows the FTIR spectrum of free paclitaxel. There were characteristic peaks in this spectrum: O–H stretching and N–H stretching in 2° amine at 3445 cm^−1^, –CH3 asymmetric and symmetric stretching at 2923 cm^−1^, conjugation of C=O with phenyl group at 1733 cm^−1^, C–O stretch at 1122 cm^−1^, and the C–H out-of-plane bending vibrations for monosubstituted rings in the paclitaxel molecule in the region of 900–500 cm^−1^.

Figure 4b demonstrates the FTIR spectrum of free curcumin. The bands exhibited in this spectrum can be assigned to: C–H stretching and O–H stretching at 3507 cm^−1^, aromatic ring C=C stretching at 1506 cm^−1^, C=O stretch at 1152 cm^−1^, and the C–H out-of-plane bending vibrations for ortho-disubstituted rings in curcumin molecule in the region of 800–600 cm^−1^.

The FTIR pattern for blank niosome (Fig. 4c) demonstrates various characteristic peaks of DOTAP, Tween-60, cholesterol, and DSPE-mPEG in the range of 3500–1115 cm^−1^. The band observed at 3435 cm^−1^ was assigned to cholesterol and Tween-60 (O–H stretching in phenols and N–H stretching in 2°-amines). C–N stretch and C–O stretch occur at 1148 cm^−1^ and belonged to DOTAP and Tween-60, respectively. The carbonyl group exhibits a strong absorption band at 1642.15 cm^−1^ due to C–O stretching vibration in DSPE-mPEG, Tween-60, and DOTAP. All peaks were repeated in the FTIR spectrum of PTX/CUR niosome formulations. The niosomal paclitaxel FTIR spectrum (Fig. 1d) shows the out-of-plane bending peaks in the range of 900–500 cm^−1^, and it can be used to assign mono-substitution on the paclitaxel ring that confirms paclitaxel loading in the niosome formulation. Furthermore, according to the niosomal curcumin FTIR spectrum (Fig. 1e), the out-of-plane bending peaks in the 800–600 cm^−1^ range can be utilized to allocate ortho substitutions on the curcumin ring that corroborates curcumin loading in the niosome formulation. When compared to the blank noisome, the sharper band in the 1600 cm^−1^ region and the broader bands in the 3500 cm^−1^ and 900–500 cm^−1^ regions in the CUR/PTX niosomal formulations (Fig. 1f, g) affirm curcumin and paclitaxel entrapment in the nano-niosomes.

### Physical stability examination

To determine physical stability, the optimum formulation of curcumin/paclitaxel-loaded niosomes, in terms of encapsulation efficiency, vesicle size, PDI, and zeta potential, were tested by storing them at 4 °C. After storage for 60 days, the encapsulation efficiency, vesicle size, PDI, and zeta potential of the optimized formulation (F7) were not significantly changed from the freshly prepared samples (p value < 0.05). These results confirmed the stability of the F7 formula.

### Cytotoxicity assays

#### IC_50s_ for individual curcumin and paclitaxel on MCF-7 and MCF-10A cells

**Fig. 5:**
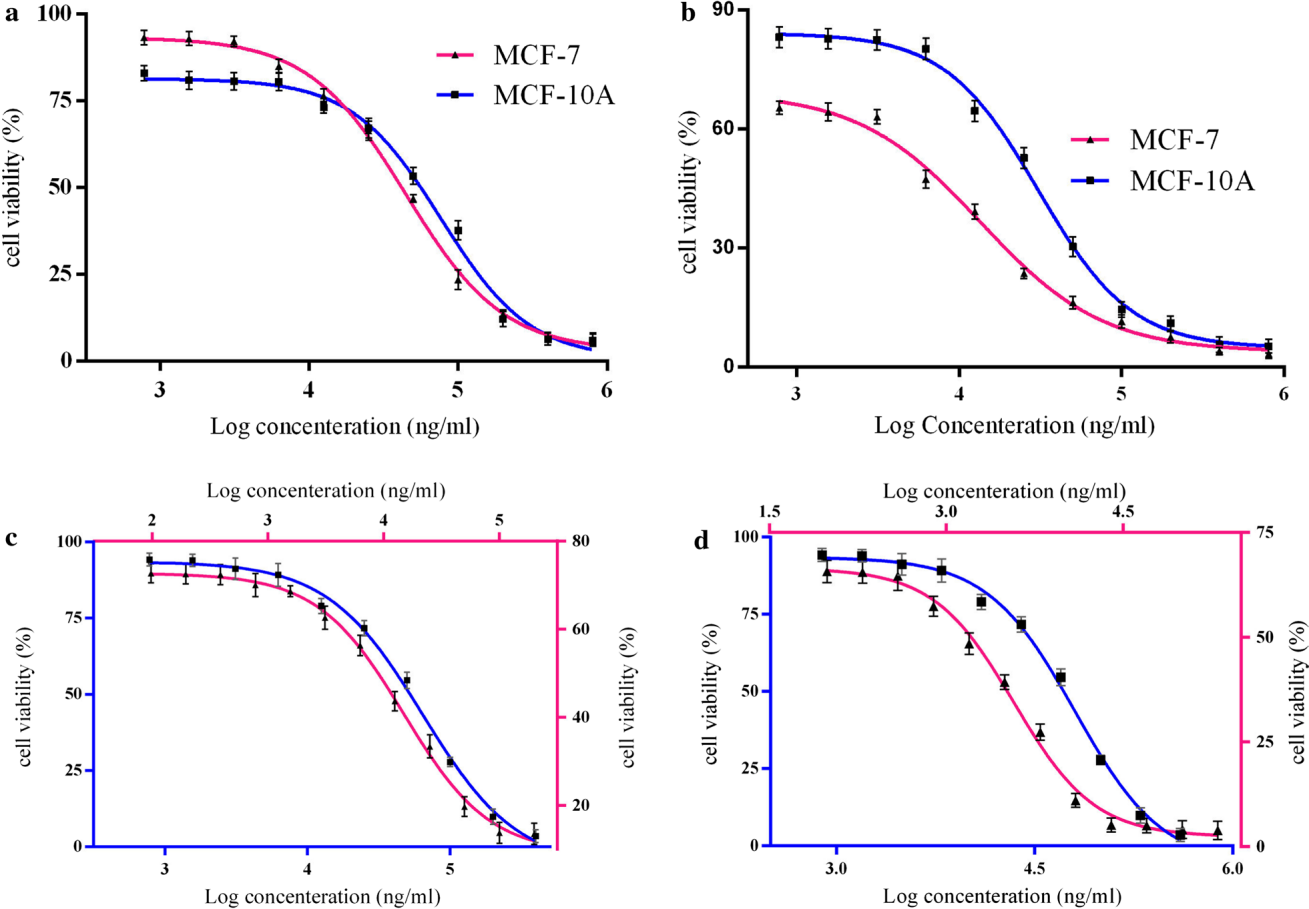
Inhibition of cell growth by curcumin (CUR) and paclitaxel (PTX) individual as a drug free form and drug niosomal form in MCF-7 and MCF-10A cell. **a** Free CUR; **b** Free PTX; **c** Nio CUR; **d** Nio PTX for MCF-7 (filled square) and MCF-10A (filled triangle) cells

To determine the inhibitory effect of individual curcumin and paclitaxel as a free form and as a niosomal form on MCF-7 and MCF-10A cells, we first performed dose–response experiments for curcumin and paclitaxel. As indicated in Fig. 5, individual treatments with the free form and the niosomal form resulted in growth inhibition of MCF-7 and MCF-10A cells in a dose-dependent pattern. Table 4 evaluates the IC_50_ values of these agents. The IC_50_ values of free PTX solution and free CUR solution was 13.54 and 44.60 μg mL^−1^, respectively, against MCF-7 cells and 30.75 and 76.71 μg mL^−1^, respectively, against MCF-10A cells (Fig. 5a, b). This revealed that MCF-10A cells needed at least a ∼ 2.27-fold higher concentration of PTX solution and a ∼ 1.7-fold higher concentration of CUR solution to attain IC_50_ compared to their counterpart MCF-7 cancer cells. As depicted in Fig. 5c, d, nano-niosomes were highly efficient in delivering the PTX and CUR drugs to both MCF-7 and MCF-10A cells. A threefold and 3.6-fold reduction in CUR and PTX concentration were measured, respectively, when the CUR and PTX were administered in nano-niosomes compared to free CUR and free PTX solutions in MCF-7 cells. Similarly, the CUR and PTX delivered in nano-niosomes to MCF-10A cells demonstrated a 1.2- and 1.5-fold lowered concentration, respectively. These results indicated that PTX and CUR in free and niosomal forms had less cytotoxicity on MCF-10A cells as a model for normal human mammary epithelial cells. The IC_50_ concentrations were then utilized to generate fixed ratios for subsequent combination experiments and for the calculation of combination index (CI).

**Table 4:** The IC50 values of paclitaxel, curcumin alone and in combination on MCF-7 and MCF-10A cells, administered in the forms of free drug and drug niosomal form

Treatment type	IC50 values	
	On MCF-7 cells	On MCF-10A cells
Free curcumin solution	44.60 ± 0.46	76.71 ± 0.45
Free paclitaxel solution	13.54 ± 0.28	30.75 ± 0.22
Free paclitaxel + free curcumin solution	8.36 ± 0.38	22.26 ± 0.48
Curcumin niosome	14.90 ± 0.19	64.22 ± 0.36
Paclitaxel niosome	3.73 ± 0.29	20.54 ± 0.49
Paclitaxel niosome + curcumin niosome	1.57 ± 0.1	8.89 ± 0.46

Data represents the mean ± SD

The values are shown as mean ± SD, n = 4

#### Growth inhibitory effects of paclitaxel in combination with curcumin

**Fig. 6:**
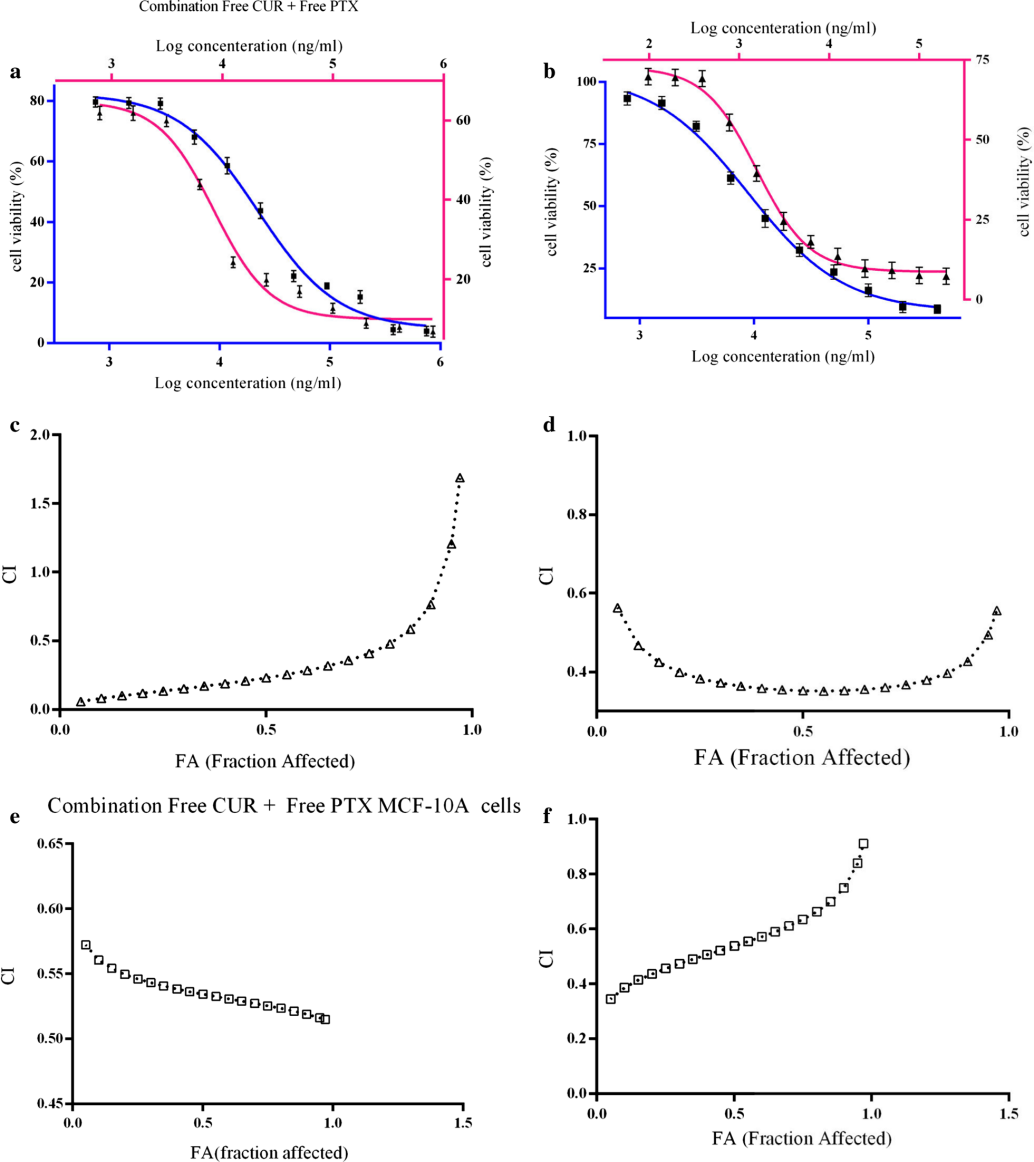
Analysis of synergy between curcumin and paclitaxel for MCF-7 (filled triangle) and MCF-10A (filled square) cells. **a** Dose–response curve of free CUR + Free PTX; **b** dose–response curve of Nio CUR + Nio PTX. CI values at different levels of growth inhibition effect (fraction affected, FA; **c** Free CUR + Free PTX in MCF-7 cells; **d** Nio CUR + Nio PTX in MCF-7 cells; **e** Free CUR + Free PTX in MCF-10A cells, **f** Nio CUR + Nio PTX in MCF-10A cell

To determine the synergistic antitumor effects of curcumin and paclitaxel, we performed a combination study, and the results are presented in Table 5. Figure 6a, b showed the dose–response curves for MCF-7 and MCF-10A cell lines exposed to paclitaxel and curcumin combination therapy. According to the results, curcumin could significantly increase the cell growth inhibition of paclitaxel; in the presence of free CUR solution, the IC_50_ of free PTX solution was diminished to ∼ 1.6-fold in MCF-7 cells and ∼ 1.4-fold in MCF-10A cells. This combination therapy regimen was significantly efficacious (p value < 0.05) when the PTX and CUR was delivered in nano-niosome formulations compared to a free solution (Table 4). Thus, the use of PTX and CUR together resulted in enhanced therapeutic potential. Figure 6 also illustrates the combination index analysis of the PTX and CUR interaction in MCF-7 and MCF-10A cells. Values of CI < 1 were obtained from the paclitaxel and curcumin combination in both free forms and niosomal forms for MCF-7 and MCF-10A cells, demonstrating that the two drugs interact synergistically to inhibit cell growth (Fig. 6c–f).

**Table 5:** Paclitaxel and curcumin combination index (CI) against MCF-7 and MCF-10A cells

Combination type	MCF-7 cells		MCF-10A cells	
	CI	Interaction type	CI	Interaction type
Free paclitaxel + free curcumin solution	0.23	Synergistic	0.53	Synergistic
Paclitaxel niosome + curcumin niosome	0.35	Synergistic	0.53	Synergistic

CI < 1, synergistic; CI = 1, additive; CI > 1, antagonistic

#### Nano-niosomal CUR/PTX cellular uptake experiments

**Fig. 7:**
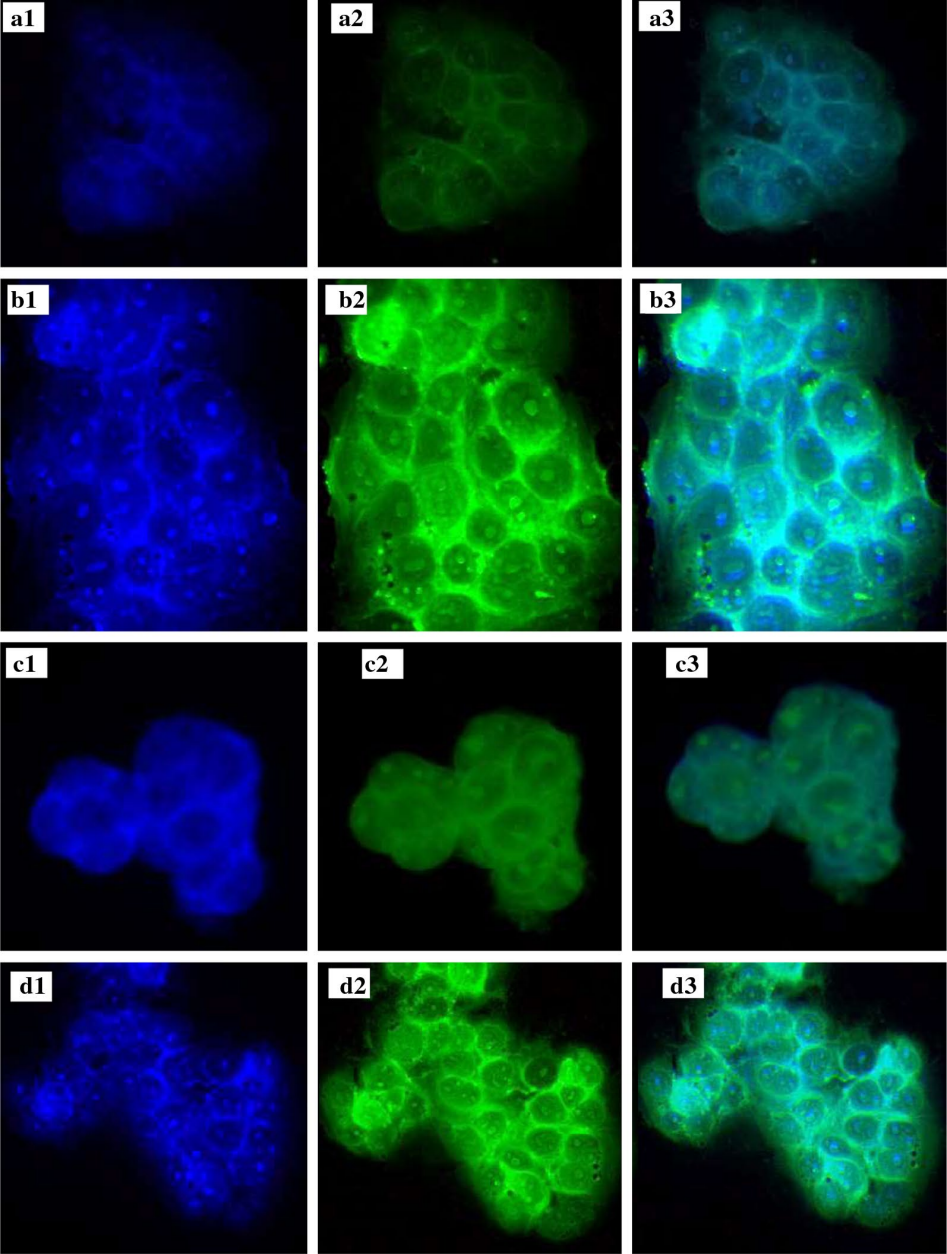
Cellular uptake of F6 and F7 CUR/PTX loaded niosomes formulations on MCF-7cell line. MCF-7cell line [**a1** F6 Nio CUR Nucleus, **a2** F6 Nio CUR, **a3** F6 Nio CUR merged; **b1** F7 Nio CUR Nucleus, **b2** F7 Nio CUR, **b3** F& Nio CUR merged; **c1** F6 PTX CUR Nucleus, **c2** F6 Nio PTX, **c3** F6 Nio CUR PTX; **d1** F7 Nio PTX Nucleus, **d2** F7 Nio PTX, **d3** F& Nio PTX merged]

**Fig. 8:**
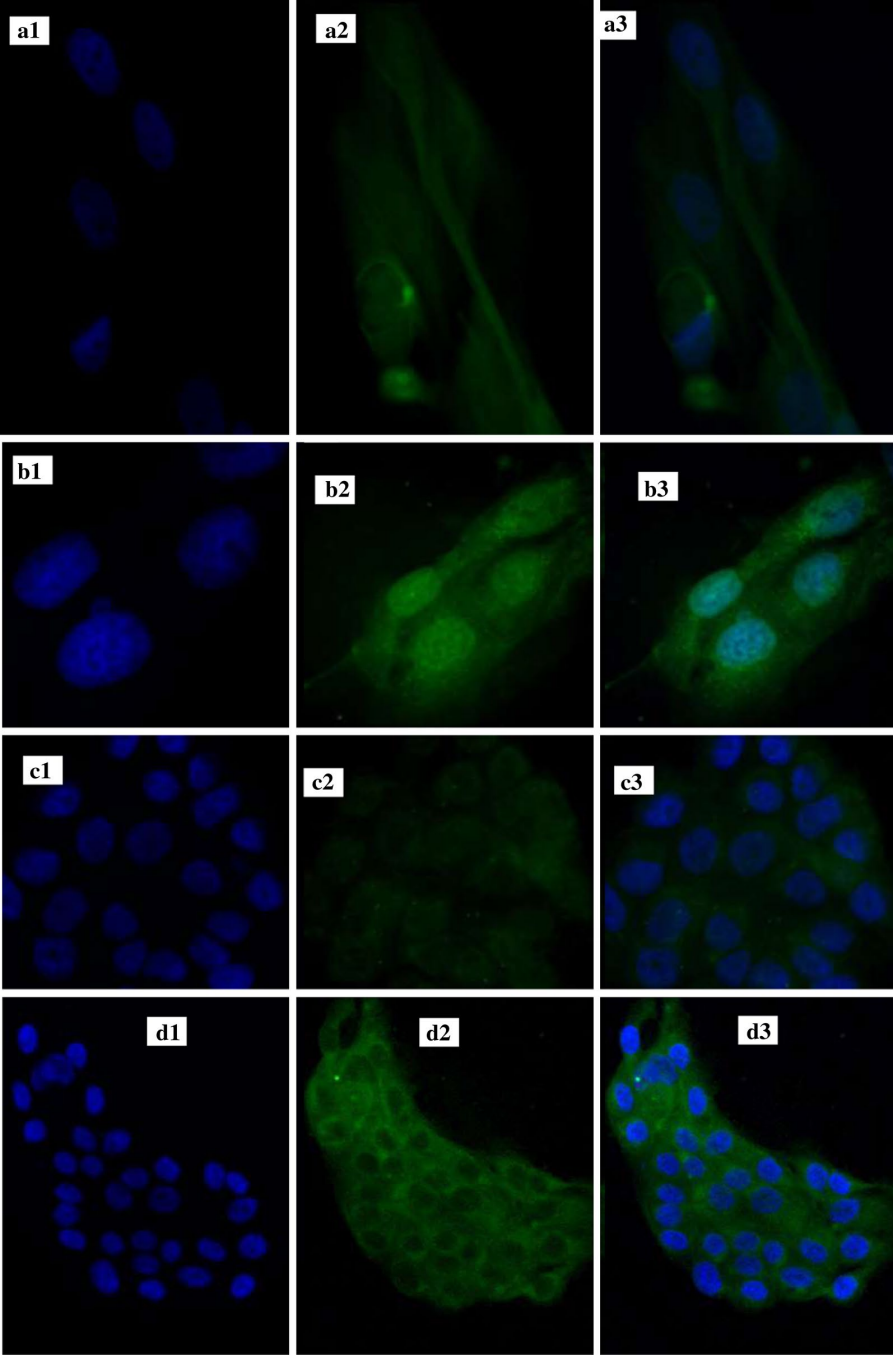
Cellular uptake of F6 and F7 CUR/PTX loaded niosomes formulations on MCF-7cell line. MCF-10A cell line [**a1** F6 Nio CUR Nucleus, **a2** F6 Nio CUR, **a3** F6 Nio CUR merged; **b1** F7 Nio CUR Nucleus, **b2** F7 Nio CUR, **b3** F& Nio CUR merged; **c1** F6 PTX CUR Nucleus, **c2** F6 Nio PTX, **c3** F6 Nio CUR PTX; **d1** F7 Nio PTX Nucleus, **d2** F7 Nio PTX, **d3** F& Nio PTX merged]

**Fig. 9:**
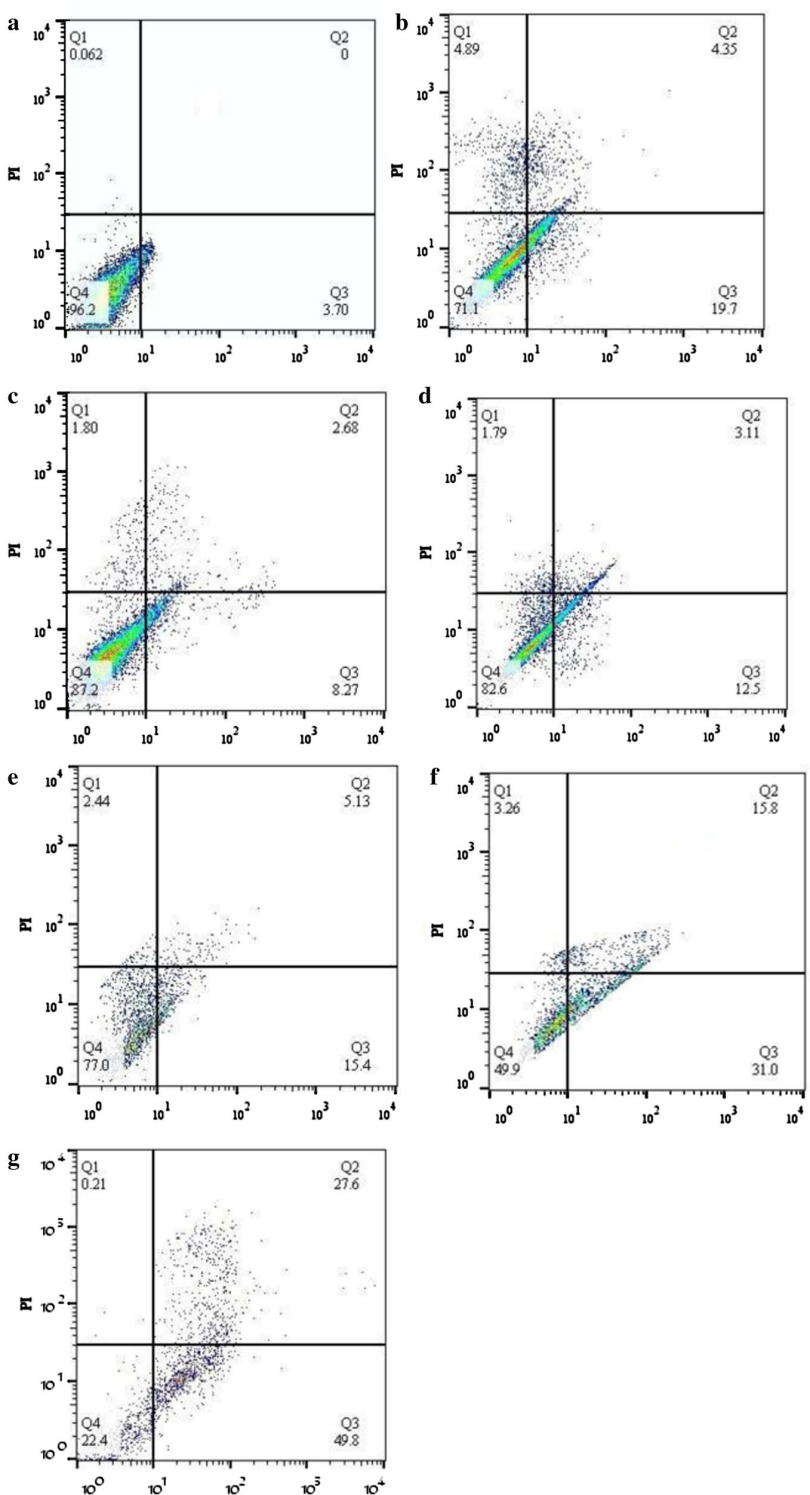
Apoptosis assay using flow cytometry following the treatment of cells for 24 h. **a** Control; **b** free curcumin + free paclitaxel; **c** free curcumin; **d** free paclitaxel; **e** niosomal curcumin; **f** niosomal paclitaxel; **g** niosomal curcumin + niosomal paclitaxel

Cellular uptake experiments were performed to evaluate the cellular uptake behavior of different CUR/PTX niosomal formulations in the following cells: MCF-7 cells as a cancer cell model and MCF10A cells as a model for normal human mammary epithelial cells. Figures 7, 8 and 9 illustrates the cellular uptake images of F6 and F7 CUR/PTX-loaded niosome formulations on MCF-7 and MCF10A cell lines monitored by fluorescence microscope. As depicted in Fig. 7b, d, the MCF-7 cells treated with the CUR/PTX F7 formula containing 10% DOTAP showed greater green and cyan (blue–green) color intensity compared to cells treated with CUR/PTX F6 formula (without DOTAP, Fig. 7a, c). By adding 10% DOTAP to the F6 formula, the drug release, vesicle size, and polydispersity index decreased, while the transfection efficiency was enhanced. Similarly, these results are observed in MCF-10A cells (Fig. 9a–d); however, the intensity of the green and cyan color in these cells was much less than in the MCF-7 cells. These findings indicate that CUR/PTX-loaded niosome formulations entered healthy cells much less than cancerous cells. These results are consistent with cytotoxicity experiments.

### Apoptosis analysis

Apoptosis was measured by annexin V-fluorescein isothiocyanate (FITC)/propidium iodide (PI) double staining (Sigma-Aldrich, USA). MCF-7 cells were seeded in six-well plates at a density of 1 × 10^5^ cells per well. Apoptosis was induced by treating the cells with PTX and CUR, either as single agents or as a PTX + CUR combination, administered in aqueous solution or in nano-niosome formulations at an IC_50_ concentration for each drug. After 24 h of incubation, the cells were detached using 0.25% trypsin/EDTA (Sigma-Aldrich, USA) and centrifuged at 1500 rpm for 3 min, after which the pellet was resuspended in ice-cold, phosphate-buffered saline (PBS, pH 7.4). Annexin V-FITC solution (3 µL) was added to each cell suspension. In addition, 3 µL of propidium iodide stock solution was added to the cells to identify necrotic cells. After 30 min of incubation on ice, the stained cells were analyzed by flow cytometry using the BD FACSCalibur instrument. Cells that did not receive any drug treatment served as the control.

## Discussion

Plants have been employed as medicines for centuries, and the usage of plant-derived chemicals has been extended into anticancer drugs. Lately, chemotherapeutic strategies have advanced to the utilization of combined active compounds because they are believed to be more active than a single agent. Hence, treatment effectiveness could increase, and the toxic side effects may be reduced, due to the extremely low use of drugs. Curcumin (diferuloylmethane), a yellow pigment isolated from the rhizome of turmeric, has been reported to have an extensive spectrum of pharmacological activities. Furthermore, curcumin is currently involved in the early phase of a clinical trial as a potential chemo-preventive agent [22, 23]. Therefore, it is logical to evaluate whether curcumin, as a new antiproliferative agent, can sensitize tumors to the chemotherapeutic drug paclitaxel for breast cancer cells. Paclitaxel (PTX) has been used as an effective chemotherapeutic drug for a wide range of tumors, such as breast, lung, prostate, ovarian, and pancreatic cancers [24, 25]. The CUR and PTX combination is a remarkable anticancer drug therapy. PTX is a powerful microtubule-stabilizing agent that commences cell cycle arrest, while CUR attacks biologically by regulating several signal transduction pathways [26–28]. Despite these good therapeutic effects, the wide therapeutic range of PTX and CUR is limited due to poor aqueous solubility and low therapeutic index. A promising approach for circumventing these issues is the use of a vesicular nanocarrier, such as niosomes, which are an alternative to phospholipid vesicles for the encapsulation of hydrophobic drugs due to providing high encapsulation efficiency, biocompatibility, biodegradation, low preparation cost, and sufficient stability, as well as being free from organic solvents and offering easy storage [7]. In this study, we have developed a novel cationic PEGylated niosomal formulation for encapsulating paclitaxel and curcumin. The vesicular systems were prepared from the nonionic surfactant Tween-60, as a commercial surfactant, and all formulations were compared in terms of entrapment efficiency, drug release, vesicle size, and polydispersity index. Furthermore, niosomes formulated without cholesterol formed a gel, and only the addition of cholesterol was a homogenous niosome obtained [29]. The hydrophilic–lipophilic balance (HLB) of the nonionic surfactant, the chemical structure of the components, and the critical packing parameter (CPP) are important in forming bilayer vesicles instead of micelles. The HLB value of a surfactant plays a key role in controlling the drug entrapment efficiency of the vesicle it forms. A surfactant, such as Tween-60, with an HLB value in the 14–17 range is inappropriate for creating niosomes. For HLB > 6, cholesterol must be added to the surfactant until forming a bilayer vesicle. Also, the presence of cholesterol in the formulation of niosomes is necessary for the physical stability of these nano-sized vesicles (i.e., suppressing the surfactant’s tendency to form aggregates, decreasing drug leakage, vesicle size, and dispersion). This was primarily ascribed to the increase in hydrophobicity (particularly with higher HLB surfactant molecules, such as Tween-60) that augmented the structural affinity of the bilayer membrane for CUR/PTX molecules [6, 11, 12, 30–32]. Therefore, cholesterol is added to the formulations as a membrane-stabilizing factor. As a result, by increasing the amount of cholesterol content from 10 to 30%, PTX/CUR entrapments in nano-niosomes were increased, while the percentage of CUR/PTX release was decreased. Furthermore, the mean diameter of niosomes increased with increasing the cholesterol content. However, the addition of cholesterol content to niosomes up to 50% decreased niosomal efficiency in trapping curcumin/paclitaxel compared to 30% cholesterol content. This finding can be explained by the possible competition between curcumin and paclitaxel as lipophilic drugs and the cholesterol incorporation into the niosomes. A further increase in cholesterol tends to deposit between the bilayers, excluding the drug from the niosomal bilayers. Above a certain level of cholesterol, entrapment efficiency decreased possibly due to a decrease in CPP [6, 11, 12, 30–32]. Improving stability, increasing the drug encapsulation, decreasing mean size diameter, and reducing drug release is due to the presence of PEGylation in the niosomal formulations [33, 34]. Therefore, 5% PEG was added to the F4 formula. According to the findings, the F6 niosomal formula demonstrated higher drug entrapment, smaller diameter, smaller PDI, and lower drug release than the F4 formula. Additionally, cationic lipids added to the niosomal formulations enhanced the niosomes’ physicochemical properties and the transfection efficiency. The addition of DOTAP decreased the drug’s release and vesicle size due to a decline in the cholesterol content. This effect also decreased the polydispersity index, which is relevant to the further reciprocal repel force between the particles with the same sign charge in the suspension system [35–38]. To hamper the aggregation of vesicular systems, it is essential to introduce a charge on the surface of the vesicle. A good indicator for the size of this barrier is zeta potential. If all the particles possess large enough zeta potential, they presumably repel each other strongly enough that they will not have the tendency to aggregate [39]. After storage for 60 days, the presence of DOTAP and PEG in niosomal formulations of CUR and PTX demonstrated no significant changes when compared to freshly prepared samples in terms of encapsulation efficiency, vesicle size, PDI, and zeta potential of the optimized formulation (F7). This implies that the new F7 niosome formulation could minimize problems associated with niosome instability, including aggregation, fusion, and drug leakage. The rate of drug release from a delivery system is a crucial factor and must be appraised to attain an optimal system with the desired drug-release profile. The in vitro release study was conducted to predict how a delivery system may function under the ideal status, which might display some indication of its in vivo efficiency. In-vitro drug release demonstrated that the cumulative release profile of CUR and PTX were apparently biphasic, with an initial rapid release period followed by a slower release phase. Because CUR and PTX are small molecules and the permeability cut-off of the dialysis bag was 12 kDa, the released CUR and PTX poured easily from the bag. As a result, neither the dialysis bag nor the drug size restricted the drug’s release. The initial fast rate of release was regulated by the diffuse mechanism (concentration gradient of CUR/PTX between noisome and buffer), while the later slow release resulted from the drug’s sustained release from the inner layer [40–42].

The in vitro release of CUR and PTX from the niosomal formulation was assessed by fitting the cumulative drug release into mathematical release models, which are commonly applied to elucidate release kinetics and to compare release profiles. The CUR/PTX niosomal formulations followed the Higuchi model. These findings indicated that CUR and PTX molecules were diffused in the niosome matrix and that there were no possible interactions between the niosome components and the drugs [5, 43–45]. In this study, we have investigated the effect of PTX and CUR combination therapy, in both free forms and niosomal forms, on MCF-7 cells as a cancer cell model and MCF10A cells as a model for normal human mammary epithelial cells (Tables 4 and 5). The ratiometric combination of PTX and CUR significantly suppressed the growth of MCF-7 cells. When the free drugs were administered in nano-niosome formulations, the cytotoxicity effects manifested even more. The enhanced therapeutic activity achieved with the combination therapy was ascribed to the P-glycoprotein (P-gp) downregulation and to the inhibition of the NFκB pathway by CUR. Most importantly, CUR downregulates the NF-ĸB signaling pathways, thus inhibiting cancer cell growth and inducing apoptosis. Therefore, CUR sensitizes cancer cells to increase the cancer cells’ response to anticancer drugs. Increasing the accumulation of PTX within the cancer cell due to P-gp downregulation can overcome the MDR phenomenon [1, 27, 46]. We observed a similar trend for MCF-10a cells. Nevertheless, as expected, CUR and PTX had fewer side effects in both free form and niosomal form on MCF10A human mammary epithelial cells. The cellular uptake experiments were demonstrated by the addition of DOTAP, which enhanced the transfection efficiency of the CUR/PTX F7 formula; it is well known that cationic lipids enhance the transfection efficiency of niosomal formulations [35–38]. Quantitative apoptotic activity measurements were made by flow cytometry analysis in PTX and CUR treated cells. Statistically significant when apoptotic activity of paclitaxel NanoNiosome formulation is compared with free paclitaxel and curcumin NanoNiosome formulation is compared with free curcumin solution in MCF-7 cells (p < 0.05). In addition to these findings, flow cytometry analysis also revealed that the apoptosis was significantly greater with the combination therapy and with drugs administered NanoNiosome formulations at p < 0.05. These results collaborate with the cell viability experiment to affirm that NanoNiosomes were effective in delivering the PTX and CUR to the cells, and combination therapy with PTX and CUR delivered in NanoNiosome formulations indeed demonstrated higher therapeutic efficacy in MCF-7 cells.

## Conclusions

Our successful findings suggest novel cationic PEGylated niosomal formulations for paclitaxel and curcumin co-administration. The encapsulation efficiency of both drugs was extremely successful. The drugs’ release profile demonstrated burst release followed by a sustained drug release for both agents. The combination of PTX (a powerful anticancer drug) with CUR (an effective chemosensitizer), particularly in nano-niosome formulations, can improve the therapeutic effectiveness of cancer treatments. Our experimental evidence indicated that a nanocarrier-based approach adopted for the delivery of CUR/PTX combinations was efficient in battling cancer cells in vitro.

## Methods

### CUR/PTX niosomes preparation

We used the thin-film hydration method to prepare the curcumin and paclitaxel-loaded niosomes [47]. Tween-60 (DaeJung Chemicals & Metals, South Korea) and cholesterol (Sigma-Aldrich, USA) were dissolved in chloroform to obtain the different molar ratio molarities (as illustrated in Table 1). PTX (Stragen, Switzerland) and CUR (Sigma-Aldrich, USA) were dissolved in chloroform and added to the mixture of surfactant and lipids. Fluorescent label Dil (Sigma, USA) was added to the lipid phase at 0.1% mol for lipid staining to evaluate cellular uptake. Niosomal formulations were screened for particle size, controlled release, and high entrapment efficiency parameters. After attaining optimized synthetic conditions, the cationic lipid DOTAP (1,2-dioleoyl-3-trimethylammonium-propane, Sigma-Aldrich, USA) and polyethylene glycol (Lipoid PE 18:0/18:0–PEG2000, DSPE-mPEG 2000, Lipoid GmbH, Germany) were added for improving stability and transfection efficiency of the niosomal formulations. Organic solvent was removed by rotary evaporator (Heidolph, Germany) at 50 °C until a thin-layered film formed. The dry lipid films were hydrated by adding phosphate-buffered saline (PBS, pH = 7.4) at 60 °C for 60 min to obtain the niosomal suspensions. After hydration, the prepared vesicles were sonicated for 30 min using a microtip probe sonicator (model UP200St, Hielscher Ultrasonics GmbH, Germany) to reduce the vesicles’ mean size. Thereafter, free drugs (unloaded) were separated from niosomal vesicles using a dialysis bag diffusion technique against PBS for 1 h at 4 °C (MW = 12 kDa, Sigma-Aldrich, USA) [48]. Drug-free niosomes were produced in a similar manner without adding curcumin and paclitaxel. The dose of both drugs was 0.5 mg mL^−1^ for all formulations.

### Analysis of encapsulation efficiency

To evaluate entrapment efficiency, spectroscopic measurements were performed. The amounts of niosomal encapsulated CUR and PTX were analyzed with a UV spectro-photometer (model T80+, PG Instruments, United Kingdom) at 429 and 236 nm (ʎmax), respectively [7]. The encapsulation efficiency was determined as follows:

$${\text{Encapsulation efficiency }}\left( \% \right) \, = \frac{{{\text{The amount of CUR}}/{\text{PTX encapsulated within niosomes}}}}{{{\text{Total amount of CUR}}/{\text{PTX added}}}} \times 100$$

### Physical characterization of niosomal vesicles

The particle size distribution, zeta potential and Poly-Dispersity Index (PDI) of the obtained niosomes were measured by dynamic light scattering technique using a ZetaPALS zeta potential and particle size analyzer (Brookhaven Instruments, Holtsville, NY, USA). Scattered light was detected at room temperature at an angle of 90°, and the diluted samples in 1700 µL of deionized water (0.1 mg mL^−1^) were prepared and immediately measured after preparation. All measurements were carried out three times, and their mean values were calculated. The internal structure of NanoNiosome formulations was determined by cryogenic transmission electron microscopy (FEI Tecnai 20, type Sphera, Oregon, USA) equipped with a LaB6 filament at 200 kV. A drop of NanoNiosome solution was placed over a 200-mesh Copper-coated TEM grids, and TEM measurement was accomplished. Characterization of surface morphology of Niosomes was evaluated using scanning electron microscope (SEM. To prepare the sample used in SEM, a little amount of the NanoNiosome solution dispersed in water was placed on the mesh copper grid 400. Then, the copper grid was placed in an evacuated desiccator to evaporate the solvent. Finally the samples were coated with gold coater to make them conductive, followed by evaluation of the surface morphology using SEM with 100 W power instrument (model KYKY-EM3200-30 kV, China).

### In-vitro drug release study

The in vitro release of CUR/PTX from niosomes was monitored using a dialysis bag (MW = 12 kDa) against PBS (containing 2% Tween-20 to imitate a physiological environment) for 72 h at 37 °C and 7.4 pH [42]. First, the CUR/PTX niosome samples were suspended in a dialysis tube, and the release of both drugs was evaluated in 10 mL of PBS with continuous stirring. Then, 2 mL of the sample was collected from the incubation medium at specific time intervals and immediately substituted with an equal volume of fresh PBS. The amount of CUR/PTX released was determined using a UV–Vis spectrometer at 429 and 236 nm, respectively.

### Mathematical modeling of drug release kinetic

Cumulative percentages of the drug released from the niosomes were calculated by the following Eq. (1):

1 $${\text{Release}} = \frac{{{\text{M}}_{\text{t}} }}{{{\text{M}}_{\text{f}} }}$$

where M_t_ and M_f_ are the cumulative amounts of drug released at any time (t) and the final amounts of drug released, respectively.

To determine the release kinetic, the release data were fitted to the mathematical models by the linear regression analysis of Graph pad prism 6.0, as follows:

Zero-order rate equation:

2 $$Q_{t} = Q_{0} + K_{0} t$$

where Q_t_ is the amount of the remaining drug in the formulation at time *t*; Q_0_ is the initial amount of drug in the formulation; and K_0_ is the zero-order release constant.

First-order rate equation:

3 $$\log {\text{C}} = \log {\text{C}}_{0 } - \frac{{{\text{K}}_{\text{t}} }}{2.303}$$

where C_0_ is the initial drug concentration; *K* is the first-order release constant; and *t* is time.

Higuchi’s model:

4 $${\text{Q}} = {\text{ K}}_{\text{H}} {\text{t}}^{ 1/ 2}$$

where Q is the amount of drug released in time *t* per unit area, and K_H_ is the Higuchi dissolution constant.

Hixson–Crowell model:

5 $${\text{Q}}_{0}^{ 1/ 3} - {\text{ Q}}_{\text{t}}^{ 1/ 3} = {\text{ K}}_{\text{s}} {\text{t}}$$

Q_0_ is the initial amount of the drug in the niosomes; Q_t_ is the cumulative amount of the drug released at time *t*; and K_s_ is the Hixson–Crowell release constant.

Finally, the correlation coefficients’ values were compared to determine the release model that best fits the data [40, 42].

### Fourier transforms infrared (FTIR) spectral evaluation

The samples’ functional group characterizations were investigated using FTIR spectrometer (Model 8300, Shimadzu Corporation, Tokyo, Japan) for pure CUR, pure PTX, blank noisome, niosomal-CUR, and niosomal-PTX. For preparation, the samples were lyophilized as a dry powder and mixed with potassium bromide (KBr). Then, the samples were placed in a hydraulic press to form the pellets. The FTIR spectrum was scanned in the wavelength range of 400–4000 cm^−1^.

### Physical stability examination

To determine the physical stability of niosomal curcumin/paclitaxel during storage, the change in particle size, zeta potential, PDI, and the remaining amount of the drug in vesicle was assessed over 14-, 28-, and 60-day intervals [9, 39].

### Cell lines and culture conditions

Human breast cancer MCF-7 cells (the Iranian Biological Resource Center, Tehran, Iran) were cultured in DMEM/F12 Ham’s mixture (InoClon, Iran) supplemented with 2 mM GlutaMAX™-I (100X, Gibco, USA), 10% FBS (Fetal Bovine Serum, Gibco, USA), and 1 mg mL^−1^ penicillin/streptomycin (Gibco, USA). Non-tumorigenic human breast epithelial cell line MCF-10A (the Iranian Biological Resource Center, Tehran, Iran) was grown in DMEM/F12 Ham’s mixture supplemented with 2 mM GlutaMAX™-I, 5% horse serum (Gibco, USA), EGF (Epithelial growth factor, Sigma, USA) 20 ng mL^−1^, insulin 10 μg mL^−1^ (Sigma, USA), hydrocortisone 0.5 μg mL^−1^ (Sigma, USA), 100 ng mL^−1^ cholera toxin (Sigma, USA), and 1 mg mL^−1^ penicillin/streptomycin. An MCF-10A cell line was used for comparison in all experiments.

### Cytotoxicity assays

The cytotoxicity of various formulations was determined by MTT (Sigma, USA) assay [49–51]. Briefly, MCF-7 and MCF-10A cells were seeded in 96-well plates at 10,000 cells per well. Following attachment for 24 h, the cells were treated with 200 μL fresh medium containing serial dilutions of the different drug/niosome formulations: free-PTX solution, free-CUR solution, free PTX + free CUR physical mixture, niosomal CUR, niosomal PCT, and the co-administration of niosomal CUR-niosomal PTX. After incubation for 48 h, 20 μL MTT (5 mg mL^−1^ in PBS) was added into each 96-well plate and incubated for 3 h at 37 °C. Finally, the medium was carefully removed, and 180 μL of DMSO was added to each well to dissolve the formazan crystals formed. Absorbance of each well was recorded by EPOCH Microplate Spectrophotometer (synergy HTX, BioTek, USA) at 570 nm. The cytotoxicity of the different formulations was expressed as the Inhibitory Concentration (IC_50_) value, defined as the drug concentration required for inhibiting cell growth by 50% relative to the control. The IC_50_ values of PTX and CUR as single drugs or in combination were calculated using GraphPad Prism 6. The curcumin and paclitaxel combination was appraised by calculating the CI value using the CompuSyn software, with the method utilized by Chou and Talalay:

6 $${\text{CI }} = \frac{a}{A} + \frac{b}{B}$$

where *a* is the PTX IC_50_ in combination with CUR at concentration *b*; *A* is the PTX IC_50_ without CUR; and *B* is the CUR IC_50_ in the absence of PTX. According to the Chou and Talalay equation, when CI < 1, the interaction between the two drugs is synergistic; when CI = 1, the interaction between the two drugs is additive; and when CI > 1, the two drugs are antagonistic [52–54].

### Nano-niosomal CUR/PTX cellular uptake experiments

MCF-7 and MCF-10A cells were seeded at a density of 2 × 10^5^ cells per well in a 6-well plate and incubated for 24 h to allow them to attach. The cells were then treated with the different NioCUR and NioPTX formulations. After 3 h of incubation, the cells were washed three times with cold PBS and fixed with a 4% paraformaldehyde solution (Sigma, USA). Then, the cells were stained with DAPI (0.125 µg mL^−1^, Thermo Fisher Scientific, USA) and imaged with a fluorescence microscope (BX61, Olympus, Japan) [48, 49, 51].

### Apoptosis analysis

An annexin V-FITC/PI double staining assay was carried out to confirm whether apoptosis was induced by curcumin or paclitaxel alone or in combination when administered in an aqueous solution and nano-niosome formulation. The results in Fig. 9 show quantitative apoptotic activity in MCF-7 cells via apoptosis assay using flow cytometry following the treatment of cells for 24 h. In apoptotic cells, the membrane phospholipid phosphatidylserine (PS) is translocated from the inner to the outer surface of the plasma membrane, thereby exposing PS to the external cellular environment. Annexin V is a 35–36 kDa Ca^2+^-dependent phospholipid-binding protein with high affinity for PS, and it binds to exposed apoptotic cell-surface PS. Annexin V can be conjugated to fluorochromes, such as FITC, while retaining its high affinity for PS, thus serving as a sensitive probe for the flow cytometric analysis of cells undergoing apoptosis. Furthermore, propidium iodide (PI) is a fluorescent intercalating agent that can be used as a DNA stain in flow cytometry. PI cannot pass the membrane of live cells and apoptotic cells; however, it stains dead cells, making it useful to differentiate necrotic, apoptotic, healthy, and dead cells. In the scatter plot of double variable flow cytometry, the Q4 quadrant (FITC−/PI−) shows living cells; the Q2 quadrant (FITC+/PI+) stands for late apoptotic cells; the Q3 quadrant (FITC+/PI−) represents early apoptotic cells; and the Q1 quadrant (FITC−/PI+) shows necrotic cells. The flow cytometry plots demonstrate there was enhancement in cellular apoptosis in MCF-7 cells when PTX and CUR were administered in nano-niosome formulations as compared to free drugs (p < 0.05). Furthermore, when PTX and CUR were co-administered in nano-niosome formulations, there was a significant increase in apoptosis (i.e., 15.27% early apoptosis in niosomal curcumin and 31.03% early apoptosis in niosomal paclitaxel versus 49.79% early apoptosis in niosomal curcumin + niosomal paclitaxel, p < 0.05). These results are consistent with the growth inhibitory effects of paclitaxel in combination with curcumin.

### Statistical analysis

Statistical data analyses were performed via GraphPad Prism 6 software and expressed as mean ± SD. A Student *t* test was used when comparing two independent groups, and an ANOVA test was used when comparing multiple samples. A p value < 0.05 was considered significant.

## Abbreviations

PTX: paclitaxel
CUR: curcumin
DDS: drug delivery system
PEG: polyethylene glycol
EE: entrapment efficiency
Cryo-TEM: cryogenic transmission electron microscopy
SEM: scanning electron microscopy
FTIR: Fourier transforms infrared
CI: combination index
